# Supplementary material for: The discovery of a novel series of compounds with single-dose efficacy against juvenile and adult Schistosoma species
Source: PLoS Negl Trop Dis. 2021 Jul 19;15(7):e0009490. doi: 10.1371/journal.pntd.0009490 (PMC8321398; doi:10.1371/journal.pntd.0009490)
Supplement: S1 Text — A detailed description of the synthesis and characterisation of LSHTM-3642 and the intermediates leading to it from commercial starting materials. (DOCX) [file pntd.0009490.s001.docx]

**S1 Text. Detailed description of the synthesis of compound LSHTM-3642**

Synthesis of **4-fluoro-1-(tetrahydro-2H-pyran-2-yl)-5-(4,4,5,5-tetramethyl-1,3,2-dioxaborolan-2-yl)-1H-indazole**

**Scheme 1**

i) NBS/MeCN, 10°C, 3 h. ii) AcOH/NaNO_2,_ 10°C to rt, 16 h. iii) 3,4-dihydropyran, PTSA, DCM, rt, 16 h. iv) bis(pinacolato)diboron, Pd(dppf)Cl_2_.DCM, KOAc, dioxan, 90°C, 16 h.

**Step i)** **Preparation of compound (II): 4-bromo-3-fluoro-2-methylaniline**

**Procedure:** To a stirred solution of 3-fluoro-2-methylaniline (300 g, 2.40 mol, 1.0 eq) in MeCN (6.0 L, 20 Vol) was added N-bromo succinimide (468 g, 2.63 mol, 1.1 eq.) portion wise at 10°C and the reaction stirred at room temperature for 3 h. The reaction mixture was evaporated under reduced pressure, the residue diluted with ethyl acetate (10 vol) and quenched with saturated aq. Na_2_S_2_O_3_ (10 vol) at 10°C. The aqueous solution was extracted with EtOAc (2 X 10 vol), the combined organic layer was washed with NaHCO_3_ solution, water and brine, dried over anhydrous Na_2_SO_4_ and evaporated under reduced pressure to afford **450 g** of crude compound (II), 4-bromo-3-fluoro-2-methylaniline, as a greyish solid, with chemical purity 70% by HPLC.

^1^H NMR (400 MHz, DMSO-d_6_) δ 7.09 (t, *J*=8.2, 1H), 6.40 (d, *J*=8.52, 1H), 1.98 (s, 3H)

LCMS rt 9.55 min, m/z = 204.1 [M+H]^+^ (Monoisotopic mass 203). Column ID: GEMINI -C18 (100X4.6) 3u, Diluent:DMSO

A:ACN B: 10mM NH_4_OAc in water; Flow:1.0ml/min, Inj. Vol. : 0.2 μl

**Step ii): Preparation of (III): 5-bromo-4-fluoro-1H-indazole**

**Procedure:** To a stirred solution of 4-bromo-3-fluoro-2-methylaniline (II), (330.0 g, 1.62 mol, 1.0 eq.) in acetic acid (4.6 L, 14 Vol) was added sodium nitrite (167.3 g, 1.97 mol, 1.5 eq.) portion wise at 10°C and the reaction mixture was stirred at room temperature for 3 h. Aqueous NaOH solution [3.5 kg, 50% W/W] was added to the reaction at -10°C, drop wise with vigorous stirring until pH was ~8-9. The mixture was extracted with ethyl acetate (3 X 10 vol), the combined organic layer was washed with brine, dried over Na_2_SO_4_ and concentrated under reduced pressure to provide the crude compound. This was purified by column chromatography over 100-200 mesh silica gel eluting with (50-60% ethyl acetate/hexane) to afford 312 g **(90% yield)** of compound (III) 5-bromo-4-fluoro-1H-indazole, as a reddish solid with chemical purity 82% by HPLC.

^1^H NMR (CDCl_3_, 400 MHz) δ: 7.19 (d, *J*=8.76, 1H), 7.46-7.49 (m, 1H), 8.14 (s, 1H), 10.50 (br s, 1H).

LCMS rt 7.54 min, m/z = 215.2 [M+H]^+^ (Monoisotopic mass: 214), Column Name: GEMINI NX( 100 X 4.6 mm ),3μ, Diluent : METHANOL, Mobile phase: -A : 0.05% HCOOH IN H2O, -B : CAN, Inj. Vol. : 0.5 μl

**Step iii) Preparation of (IV): 5-bromo-4-fluoro-1-(tetrahydro-2H-pyran-2-yl)-1H-indazole**

**Procedure:** To a stirred solution of (150.0 g, 697.7 mmol, 1.0 eq.) in dichloromethane (4.5 L, 30 Vol] was added 3,4-dihydropyran (126 mL, 1.13 mol, 2.0 eq.) followed by PTSA (11.9 g, 62.6 mmol, 0.1 eq.) and the reaction mixture stirred at 25°C for 3 h under N_2_. The reaction mixture was quenched with NaHCO_3_ solution (10 Vol) and washed with water (5 Vol) then brine [5 Vol]. The organic layer was dried over anhydrous Na_2_SO_4_ and evaporated under reduced pressure to provide crude compound. This was purified by column chromatography over 100-200 mesh silica gel eluting with (5% ethyl acetate in hexane), to give 110 g of compound (IV), 5-bromo-4-fluoro-1-(tetrahydro-2H-pyran-2-yl)-1H-indazole  **(53% yield)** as a reddish solid.

^1^H NMR (CDCl_3_, 400 MHz) δ: 1.70-1.78 (m, 3H), 2.06-2.14 (m, 2H), 2.45-2.54 (m, 1H), 3.69-3.75 (m, 1H), 3.93-3.99 (m, 1H), 5.68 (dd, 1H), 7.25-7.29 (m, 1H), 7.43-7.47 (m, 1H), 8.06 (s, 1H).

LCMS m/z = 299.2 [M+H]^+^ (Monoisotopic mass 298)

**Step iv) Preparation of (V): 4-fluoro-1-(tetrahydro-2H-pyran-2-yl)-5-(4,4,5,5-tetramethyl-1,3,2-dioxaborolan-2-yl)-1H-indazole**

**Procedure:** To a stirred solution of 5-bromo-4-fluoro-1H-indazole (IV), (350 g, 1.11 mol, 1.0 eq.) in 1,4-dioxan (7.0 L, 20 Vol) was added bis(pinacolato)diboron (595 g, 2.34 mol, 2.0 eq.) followed by potassium acetate (345 g, 3.33 mol, 3.0 eq.). The reaction mixture was degassed with Ar_(g)_, then Pd (dppf)Cl_2_.DCM (96 g, 117.6 mmol, 0.1 eq.) added and the mixture degassed for a further 15 minutes. The reaction was stirred at 90°C for 16 h under N_2_. The cooled reaction was filtered through Celite, washing through with ethyl acetate. The filtrate was concentrated under reduced pressure, water (10 vol) was added and the aqueous extracted with ethyl acetate (3 X 10 vol). The combined organic layer was washed with water (5 Vol) then brine (5 Vol) and dried over Na_2_SO_4_ and evaporated under reduced pressure. The crude product was purified by column chromatography over 100-200 mesh silica gel eluting at 5% ethyl acetate in hexane. The required fractions were collected and distilled under reduced pressure to afford 220 g **(54% yield)** of compound (V), 4-fluoro-1-(tetrahydro-2H-pyran-2-yl)-5-(4,4,5,5-tetramethyl-1,3,2-dioxaborolan-2-yl)-1H-indazole, as greenish liquid which solidified on standing.

^1^H NMR (CDCl_3_, 400 MHz) δ: 1.37 (s, 12H), 1.60-1.77 (m, 3H), 2.03-2.07 (m, 1H), 2.13-2.15 (m, 1H), 2.52-2.54 (m, 1H), 3.71-3.75 (m, 1H), 3.99-4.01 (m, 1H), 5.69 (dd, 1H), 7.31 (d, *J*=8.56, 1H), 7.65-7.69 (m, 1H), 8.09 (s, 1H).

LCMS rt 8.12 min, m/z = 347.3 [M+H]^+^ Column Name: GEMINI NX( 100 X 4.6 mm ),3μL, Diluent: METHANOL, Mobile phase: -A : 0.05% HCOOH IN H2O, -B : CAN, Inj. Vol. : 0.5 μl

**Scheme 2**

v) DMA, 80-85°C, 16 h. vi) PdCl_2_(dppf).DCM, K_3_PO_4_, dioxan-water (4:1), 90°C, 16h. vii) NBS, DCE, reflux, 16 h. viii) Pd(PPh_3_)_4_, K_3_PO_4_, dioxan-water (4:1), 90°C, 16 h. ix) 20% TFA/DCM, 1 h, rt.

**Step-v): Preparation of compound (VII), 6-bromo-2-(trifluoromethyl)imidazo[1,2-a]pyrazine:**

**Procedure:** To a stirred solution of 5-bromopyrazin-2-amine (100 g, 575 mmol, 1.0 eq.) in DMA (1.0 L, 10 vol) was added 3-bromo-1, 1, 1-trifluropropan-2-one (274.4 g, 1.44 mol, 2.5 eq.) under N_2,_ and the reaction mixture heated at 60°C for 16-20 h. After completion, the mixture was cooled to room temperature and carefully poured into saturated NaHCO_3_ solution (15 vol.) (care: effervescence). The mixture was extracted with ethyl acetate (3 X 10 vol.), the combined organic layer was washed with water and brine solution (2 vol.), dried over Na_2_SO_4_ and concentrated under reduced pressure. The crude product was purified by column chromatography over 100-200 mesh silica gel, eluting with ethyl acetate/hexane (0/100 to 100/0). The desired fractions were collected and distilled under reduced pressure to afford 75 g (49% yield) of 6-bromo-2-(trifluoromethyl)imidazo[1,2-a]pyrazine, as liquid which solidified on standing.

^1^H NMR (400 MHz, DMSO-d_6_) δ 9.13 (s, 1H) 8.99 (s, 1H), 8.64 (s, 1H).

LCMS rt 2.87 min, m/z 266.02.

**Step-vi): Preparation of compound**-(VIII): 6-(4-fluoro-3-(trifluoromethyl)phenyl)-2-(trifluoromethyl)imidazo[1,2-a]pyrazine

**Procedure** To a stirred solution 6-bromo-2-(trifluoromethyl)imidazo[1,2-a]pyrazine (VII) (50.0g, 187.95 mmol, 1.0 eq.) in dioxan:water (4:1, 1.0 L) was added (4-fluoro-3-(trifluoromethyl)phenyl)boronic acid (Commercially available, 66.17 g, 319.5 mmol, 1.7 eq.) followed by potassium phosphate (122.50 g, 563.85 mmol, 3eq.). The reaction mixture was degassed with Ar_(g)_ and [1,1'-bis(diphenylphosphino) ferrocene] palladium(II) chloride, DCM complex (15.34 g, 18.79 mmol, 0.1 eq.) was added and the reaction mixture stirred at 90°C for 16 h under N_2_. After the starting material was consumed (monitored by both LCMS and TLC), the solvent was evaporated under reduced pressure, the residue was diluted with ethyl acetate and filtered through Celite. The filtrate was washed successively with water and brine, the organic layer dried over Na_2_SO_4_, filtered and evaporated under reduced pressure. The crude compound was purified by column chromatography over 100-200 mesh silica gel, eluting with 10% ethyl acetate in hexane to give 50 g (**76% yield**) of 6-(4-fluoro-3-(trifluoromethyl)phenyl)-2-(trifluoromethyl)imidazo[1,2-a]pyrazine.

^1^H NMR (CDCl_3_, 400 MHz) δ: 7.34 (t, *J*=9.2, 1H), 8.06 (s, 1H), 8.12-8.14 (m, 1H), 8.19-8.21 (m, 1H), 8.49 (s, 1H), 9.25 (s, 1H). LCMS m/z = 350.0 [M+H]^+^

**Step-vii): Preparation of compound-(IX): 3-bromo-6-(4-fluoro-3-(trifluoromethyl)phenyl)-2-(trifluoromethyl)imidazo[1,2-a]pyrazine**

**Procedure:** To a stirred solution of 6-(4-fluoro-3-(trifluoromethyl)phenyl)-2-(trifluoromethyl)imidazo[1,2-a]pyrazine (40 g, 114.43 mmol, 1.0 eq.) in 1,2-dichloroethane (1.0 L) was added NBS (40.77 g, 229.08 mmol, 2.0 eq.) and the reaction mixture stirred at 90°C for 16 h. After complete consumption of the starting material (monitored by both TLC and LCMS) the solvent was evaporated under reduced pressure to provide crude product. This was purified by column chromatography over 100-200 mesh silica gel eluting with: 10% ethyl acetate in DCM to give 40 g (**81 % yield**) of 3-bromo-6-(4-fluoro-3-(trifluoromethyl)phenyl)-2-(trifluoromethyl)imidazo[1,2-a]pyrazine.

^1^H NMR (CDCl_3_, 400 MHz) δ: 7.37 (t, 1H), 8.17-8.19 (m, 1H), 8.26-8.28 (m, 1H), 8.43 (s, 1H), 9.22 (s, 1H). LCMS m/z = 428 [M+H]^+^

**Step-viii): Preparation of compound-(X): 3-(4-fluoro-1-(tetrahydro-2H-pyran-2-yl)-1H-indazol-5-yl)-6-(4-fluoro-3-(trifluoromethyl)phenyl)-2-(trifluoromethyl)imidazo[1,2-a]pyrazine**

**Procedure:** To a stirred solution 3-bromo-6-(4-fluoro-3-(trifluoromethyl)phenyl)-2-(trifluoromethyl)imidazo[1,2-a]pyrazine (40.0 g, 93.45 mmol, 1.0 eq.) in 1,4-dioxan:water (4:1, 1.8 L) was added 4-fluoro-1-(tetrahydro-2H-pyran-2-yl)-5-(4,4,5,5-tetramethyl-1,3,2-dioxaborolan-2-yl)-1H-indazole, compound (V), (64.02 g, 185.04 mmol, 2.0 eq.) followed by potassium phosphate (60.91 g, 280.35 mmol, 3 eq.). The reaction mixture was degassed with Ar_(g)_ for 15 minutes and Pd(PPh_3_)_4_ (10.79 g, 9.34 mmol, 0.05 eq.) was added and the reaction stirred at 80°C for 16 h under N_2_. After complete consumption of the starting material (monitored by both LCMS and TLC) the solvent was evaporated under reduced pressure, the residue was diluted with ethyl acetate and filtered through Celite. The filtrate was washed successively with water and brine, the organic layer dried over Na_2_SO_4_, filtered and evaporated under reduced pressure to provide crude compound. This was purified by column chromatography over 100-200 mesh silica gel, eluting with 10% ethyl acetate in hexane to give the 32 g (**60.4% yield**) of 3-(4-fluoro-1-(tetrahydro-2H-pyran-2-yl)-1H-indazol-5-yl)-6-(4-fluoro-3-(trifluoromethyl)phenyl)-2-(trifluoromethyl)imidazo[1,2-a]pyrazine.

^1^H NMR (CDCl_3_, 400 MHz) δ: 1.72-1.81 (m, 3H), 2.18-2.20 (m, 2H), 2.57-2.61 (m, 1H), 3.78-3.83 (m, 1H), 4.07-4.14 (m, 1H), 5.81 (t, 1H), 7.27-7.30 (m, 1H), 7.40-7.43 (m, 1H), 7.61-7.68 (m, 1H), 8.01-8.04 (m, 2H), 8.15-8.18 (m, 1H), 8.24 (d, 1H), 9.33 (d, 1H).

LCMS m/z = 568.1 [M+H]^+^

**Step-ix): Preparation of SAL 656:** **3-(4-fluoro-1H-indazol-5-yl)-6-(4-fluoro-3-(trifluoromethyl)phenyl)-2-(trifluoromethyl)imidazo[1,2-a]pyrazine**

**Procedure:** To a stirred solution of 3-(4-fluoro-1-(tetrahydro-2H-pyran-2-yl)-1H-indazol-5-yl)-6-(4-fluoro-3-(trifluoromethyl)phenyl)-2-(trifluoromethyl)imidazo[1,2-a]pyrazine (30 g, 52.8 mmol, 1.0 eq.) in ethanol (600 mL, 20 Vol) at room temperature, was added PTSA (20.10 g, 116.9 mmol, 2.0 eq.) The reaction mixture was heated at 80°C for 36-48 h under N_2_. After complete consumption of the starting material (monitored by both TLC and LCMS) the volatiles were evaporated under reduced pressure. The crude compound was diluted with water (7 vol) and extracted with DCM (3X 10 vol). The combined organic layer was washed with sodium bicarbonate solution, brine and dried over Na_2_SO_4_ and evaporated under reduced pressure. The crude product was re-crystallized from ethyl acetate and hexane, the solid was further washed with hexane and dried under reduced pressure to afford 15 g SAL 656 (**59 % yield**) 3-(4-fluoro-1H-indazol-5-yl)-6-(4-fluoro-3-(trifluoromethyl)phenyl)-2-(trifluoromethyl)imidazo[1,2-a]pyrazine, as white solid with 99.91% chemical purity by HPLC.

^1^H NMR (DMSO-d_6_, 400 MHz) δ: 7.53-7.65 (m, 3H), 8.38-8.43 (m, 3H), 8.86 (s, 1H), 9.46 (s, 1H), 13.72 (s, 1H). LCMS m/z = 484.2 [M+H]^+^ (MWt = 483.3).

^13^C NMR (DMSO-*d_6_*, 100 MHz) δ 159.05 (^1^J_CF_= 254.18 Hz), 153.31(^1^J_CF_= 252.91 Hz) , 144.1 (CH), 143.8, 138.6, 137.2, 134.2, 133.1 (CH), 132.5, 130.6 (CH), 129.0 (CH), 125.0 (CH), 123.8, 122.7, 120.5, 117.6 (CH), 116.9, 115.4 (CH), 112.8, 107.5 (CH), 101.8.

UPLC rt 3.22 min

In the PDF documents below LSHTM-3642 is referred to as SAL656

**Details of LCMS methods unless otherwise stated**

Instrument Name: LCMS/MS API 2000

Instrument manufacturer: Applied Biosystem

HPLC: Shimadzu Prominence

**Column (Name, Size, type):**

Zorbax Extend (C18 4.6 X 50 mm, 5 micron)

**Eluent (solvent):**

A channel: 10 mM Ammonium Acetate in water

B channel: Acetonitrile (Organic phase)

Dual Wavelength: At 220 and 260 nm

Detector: UV

**Gradient condition:**

A: Buffer 10 mM Ammonium Acetate in water

B: Acetonitrile

Flow rate: 1.2 ml/min

Column Temperature: 25 ºC

Injection Volume: 2 µL

**LC-MS gradient:**

mobile phase: from 90% [buffer] and 10% [CH_3_CN] to 70% [buffer] and 30% [CH_3_CN] in 1.5 min, further to 10% [buffer] and 90% [CH_3_CN] in 3.0 min, held this mobile phase composition to 4 min and finally back to initial condition in 5 min).

| TIME | MODULE | % A (Buffer) | % B (CH_3_CN) |
| --- | --- | --- | --- |
| 0.01 | Pumps | 90 | 10 |
| 1.50 | Pumps | 70 | 30 |
| 3.00 | Pumps | 10 | 90 |
| 4.00 | Pumps | 10 | 90 |
| 5.00 | Pumps | 90 | 10 |
| 5.10 | System Controller | Stop |  |

**Mass conditions:**

Ionization technique: ESI (Electron Spray Ionization) using API (Atmospheric pressure Ionization) source

Declustering Potential: 10-70 V depending on the ionization of compound

Mass range: 100-800 amu

Scan type: Q1

Polarity: + Ve

Ion Source: Turbo spray

Ion spray voltage: +5500 for +ve mode

Mass Source temperature: 200 deg C
