# Supplementary material for: The discovery of a novel series of compounds with single-dose efficacy against juvenile and adult Schistosoma species
Source: PLoS Negl Trop Dis. 2021 Jul 19;15(7):e0009490. doi: 10.1371/journal.pntd.0009490 (PMC8321398; doi:10.1371/journal.pntd.0009490)
Supplement: S1 Table — A look-up table between compounds in this paper and compounds in patent WO2018130853 [21] where the synthesis and characterisation of further examples is described. (DOCX) [file pntd.0009490.s002.docx]

**S1 Table. Look-up table between compounds described in the paper and patent** **WO2020016235 (21).**

Readers interested in the synthetic details of compounds described in the paper are directed to patent WO2020016235 where further synthetic details of most of the potent compounds are provided. The table below provides a look-up between the two documents.

| **Paper ID** | **Example number in patent** |
| --- | --- |
| 16 | 6 |
| 18 | 4 |
| 19 | 15 |
| 20 | 9 |
| 21 | 19 |
| 22 | 1 |
| LSHTM-3520 | 17 |
| 23 | 27 |
| LSHTM-3642 | 50 |
| LSHTM-3608 | 29 |
| LSHTM-3686 | 79 |
| LSHTM-3645 | 63 |
| LSHTM-3661 | 67 |
| LSHTM-3604 | 2 |
| LSHTM-3690 | 81 |
| LSHTM-3644 | 61 |
