# Supplementary material for: The discovery of a novel series of compounds with single-dose efficacy against juvenile and adult Schistosoma species
Source: PLoS Negl Trop Dis. 2021 Jul 19;15(7):e0009490. doi: 10.1371/journal.pntd.0009490 (PMC8321398; doi:10.1371/journal.pntd.0009490)
Supplement: Attachment — Submitted filename: 2021_05_12_response_to_reviewers.docx [file pntd.0009490.s003.docx]

Salvensis

Discovery Park

Ramsgate Rd

Sandwich

Kent CT13 9ND

12^th^ May 2021

Re PNTD-D-21-00141

Dear Editor PLOS NTD,

Many thanks to you & your team for reviewing our manuscript “The discovery of a novel series of compounds with single-dose efficacy against juvenile and adult Schistosoma species”. We very much appreciate the time that has been taken and the detailed comments & suggestions. We have addressed the points in the manuscript as described below.

| PLOS/reviewer’s comments | Authors response |
| --- | --- |
| I agree with all the reviewers that this manuscript represents an important contribution that identifies new compounds with therapeutic potential against schistosomes. I also agree with some of the concerns of the reviewers. The authors might consider the suggestion of Reviewer 1 to split off the medicinal chemistry section, though I would not consider that a requirement for acceptance. Most of the other concerns were minor. However, if resubmitting, I would strongly urge the authors to include page and line numbers to make reviewing less cumbersome.  Reviewer #1: The objectives are clearly described and the design is appropriate. My concern about the methods represents a weakness in the manuscript, which contains a significant component of medicinal chemistry, a feature not usually found in papers published in this journal. Methods for the synthesis of the compounds evaluated in bioassays are typically provided in detail; this is not the case here, and the authors refer to the contract lab that made them. This needs to be addressed. Indeed, although this is a very important manuscript, I believe it should be split into two (a rare recommendation, to be sure): the med them work would be better placed in a more relevant journal, with details of the syntheses and perhaps the juvenile in vitro bioassay data as the biological component. The manuscript for PLoS-NTDs should focus only on the results of the most promising compounds (and these results are truly exciting).  Reviewer #2: It would be helpful to have a fuller description of the methods used in some instances, rather than just a reference.  Reviewer #3: well done --------------------  Editorial and Data Presentation Modifications Reviewer #1: The lack of page and line numbering is an impediment for me, but I offer a few minor suggestions for improvement. 1. Abstract: although PZQ is given in a high dose, the cost of goods is low enough to permit it. Rather than refer to the size of the dose, the authors should prioritize ease of administration with a cos-of-goods compatible with eventual donation. 2.Throughout the manuscript, separate numbers from units (i.e., 4 mM instead of 4mM, etc.) 3.Last sentence of the Introduction: these are candidates for clinical development, not fir use 4. M&M: were the observers of the mice blinded to the treatment? What kinds of adverse events were they trained to look for? 5. 1st sentence, Bioanalysis of plasma samples: the meaning of "all samples were extracted using protein precipitation" is unclear; please provide missing details. 6. Results, 2nd paragraph, 7: did the imidazopyrazine actually confer greater activity, or only appear to? 7. Last sentence, legend to Fig. 5:"overall it looks as though binding is similar between mouse and human plasma"; is the binding similar or is it not? 8. First sentence after this legend: use 'human' instead of 'man'. Your meaning is clear, but this usage is now outdated 9. Last sentence in the 2nd paragraph before Fig. 7: replace 'infected by' with 'of' 10. Next sentence after this: replace 'low dose efficacy' with 'high potency' 11. I could find no mention of the adoption of this project by Merck KGaA in the citation (33) provided in the Acknowledgments. Please update/clarify.  Reviewer #2: P21 - … two most clinically relevant species S. mansoni and S. haematobium …  Testing against both adult and larval worms of all three species would be informative.  P34, Table 4, formulation column ‘F2’ an ‘F1’ should be replaced by appropriate ‘Aq’ or ‘corn oil’  Reviewer #3: (Very) Minor Revision, see below  -       M&M, Pharmacokinetics: replace 4000 rpm by 4,000 rpm. Add space between 30 and µl. -       Here and elsewhere, spaces between numbers and units are often missing, example: 10 μL of 10mM (in: Plasma Protein Binding in mouse or human plasma …), or pH7.4 on page 30. -       Use italics for species names throughout the text (see e.g. S. mansoni in the legend of Table 1). | Although we considered submitting a medicinal chemistry paper separately, we preferred one paper and consider that it would be difficult to split now; especially as the team is now engaged in other projects.  We have a word version with page & line numbers but have been unable to produce a PDF which reproduces the text correctly when page & line numbers are included. Perhaps you can point us to software that might be able to help in this regard?  We have modified the MS to make it clearer that synthetic details of the final compounds are described in the patent referenced in the paper. In addition, we have added a look-up table in supplementary information to make it much easier for the reader to find in the patent the synthetic details of specific compounds described in the paper.  More detail has been added to the first part of the section “Parasite maintenance, preparation and assay methods”.  1- The point about cost of goods for PZQ permitting a large dose is fair & perhaps we do focus on dose-size more than necessary. Nevertheless 40mg/kg is a large dose & (for example) is well above MMV’s target of 500mg for a total human dose to aid patient compliance. Modifications made to the target candidate profile in the last paragraph of the introduction.  2 – all corrections made  3 – changed to “to promising candidates for clinical development which may subsequently prove to be useful in the treatment of schistosomiasis”  4 – The fact that those observing the mice included observers blinded to the treatments and the effects they were trained to look for is addressed by revision of the methods section.  5 – Further details have been added  6 – reworded “by LSHTM-1507 and LSHTM-1945, the imidazopyrazine conferred greater activity”  7 – reworded “Despite difficulties in accurate measurement due to very high levels of protein binding, the data is consistent with equal levels of protein binding in mouse and human plasma.”  8 –changed  9 – changed  10 – changed  11 – The date in the reference was incorrect, you need to scroll through to the date 2019/09/09. As of 24^th^ March this is on page 4 of that website.  This is true. Table 3 is the only data we gathered across species. We believe Merck have been carrying out more testing in different species, results that will hopefully be published in due course.  Formulation – F1 & F2 changed as appropriate  Done  Done  done |

Best regards,

Dr. Mark Gardner

[mark.gardner@salvensis.org](about:blank)

+44 7801 561372
